# Integrated Network Pharmacology, Molecular Docking, Molecular Simulation, and In Vitro Validation Revealed the Bioactive Components in Soy-Fermented Food Products and the Underlying Mechanistic Pathways in Lung Cancer

**DOI:** 10.3390/nu15183949

**Published:** 2023-09-12

**Authors:** Abd Elmoneim O. Elkhalifa, Humera Banu, Mohammad Idreesh Khan, Syed Amir Ashraf

**Affiliations:** 1 Department of Clinical Nutrition, College of Applied Medical Sciences, University of Ha’il, Ha’il P.O. Box 2440, Saudi Arabia; 2 Department of Clinical Nutrition, College of Applied Health Sciences in Ar Rass, Qassim University, Ar Rass 51921, Saudi Arabia

**Keywords:** soybean, isoflavones, daidzein, cancer, molecular dynamic simulation, MTT assay, MMP9, IGF-1R

## Abstract

Globally, lung cancer remains one of the leading causes of cancer-related mortality, warranting the exploration of novel and effective therapeutic approaches. Soy-fermented food products have long been associated with potential health benefits, including anticancer properties. There is still a lack of understanding of the active components of these drugs as well as their underlying mechanistic pathways responsible for their anti-lung cancer effects. In this study, we have undertaken an integrated approach combining network pharmacology and molecular docking to elucidate the mechanism of action of soy-fermented food products against lung cancer through simulation and in vitro validation. Using network pharmacology, we constructed a comprehensive network of interactions between the identified isoflavones in soy-fermented food products and lung cancer-associated targets. Molecular docking was performed to predict the binding affinities of these compounds with key lung cancer-related proteins. Additionally, molecular simulation was utilized to investigate the stability of the compound–target complexes over time, providing insights into their dynamic interactions. Our results identified daidzein as a potential active component in soy-fermented food products with high binding affinities towards critical lung cancer targets. Molecular dynamic simulations confirmed the stability of the daidzein–MMP9 and daidzein–HSP90AA1 complexes, suggesting their potential as effective inhibitors. Additionally, in vitro validation experiments demonstrated that treatment with daidzein significantly inhibited cancer cell proliferation and suppressed cancer cell migration and the invasion of A549 lung cancer cells. Consequently, the estrogen signaling pathway was recognized as the pathway modulated by daidzein against lung cancer. Overall, the findings of the present study highlight the therapeutic potential of soy-fermented food products in lung cancer treatment and provide valuable insights for the development of targeted therapies using the identified bioactive compounds. Further investigation and clinical studies are warranted to validate these findings and translate them into clinical applications for improved lung cancer management.

## 1. Introduction

Millions of people around the world suffer from lung cancer, which is a serious and widespread health problem [1]. The symptoms of lung cancer include coughing, chest pain, shortness of breath, and coughing up blood. Lung cancer starts in the cells of the lungs. As well as spreading to other parts of the body, lung cancer may also cause complications and damage to the brain, liver, bones, and adrenal glands [2]. It has been reported that lung cancer is the second most common cancer in the world, with more than 2.2 million new cases and 1.8 million deaths expected by 2020, as reported by the World Health Organization (WHO) [3]. Women are more likely to develop lung cancer than men, according to the American Cancer Society [4]. Lung cancer incidence and mortality rates vary widely across regions and countries, depending on factors such as socioeconomic development, tobacco use, environmental exposure, and health system capacity [2,3]. The WHO projects that by 2040, there will be 3.5 million new cases and 2.9 million deaths from lung cancer per year [4].

The foremost cause of lung cancer is smoking, which exposes the lungs to harmful chemicals that damage the DNA of cells and make them grow abnormally [1]. Smoking accounts for about 80% to 90% of lung cancer cases [5]. However, lung cancer incidence can also occur in individuals who have never smoked or who have been exposed to second-hand smoke, asbestos, radon gas, air pollution, or other environmental causes and genetic factors [1,5]. Surgery, radiation, and chemotherapy are some of the cancer treatments developed in recent years. It is estimated that only one third of cancer patients can be cured by surgery or radiation therapy; however, in the case of cancer that has spread to other areas of the body, chemotherapy is considered a systemic treatment [6]. Various cancer treatment options could lead to major side effects and even high costs due to the likelihood of serious side effects. As a result, it is necessary for the development and finding of new medicines derived from natural sources, particularly plant-based functional foods, in order to ensure that these drugs are abundantly available as well as relatively cheaper in price [6].

Soybean-fermented foods are foods that are made from soybeans that have undergone a process of fermentation by microorganisms [7]. Soybean-fermented foods are widely consumed in Asian countries, such as China, Japan, Korea, and Indonesia, where they have been part of the traditional diet for centuries [8]. Some of the most common soybean-fermented foods are miso, tempeh, natto, soy sauce, and douchi. These foods vary in their appearance, flavour, texture, and preparation methods, but they all share some health benefits [9]. Soybean-fermented foods are rich in protein, fiber, phytochemicals, vitamins, minerals, and probiotics. They may help improve digestion, immunity, metabolism, and cardiovascular health. They may also prevent or treat some chronic diseases, such as diabetes, obesity, cancer, and osteoporosis [9,10,11,12].

There are several compounds in soybean-fermented foods that have anticancer properties, including saponins, phenolic compounds, and phytic acid, as well as enzyme inhibitors such as trypsin and Bowman–Birk inhibitors, although the most prominent compounds are isoflavones, which are powerful antioxidants that can shield human cells from oxidative stress that can lead to cancer [12,13]. Isoflavones comprise three types of aglycones, daidzein, genistein, and glycitein, and their respective glycosides, daidzin, genistin, and glycitin. Isoflavones can also exist as conjugated compounds, such as malonylglucoside, acetylglucoside, and succinylglucoside [14,15]. In soybean-fermented foods, there are a number of factors that can affect the content and profile of isoflavones. This includes the variety of soybean, the fermentation method, the microorganisms involved, the processing conditions, and the period of storage [16,17]. Genistein, an isoflavone that occupies a predominant position in soy, has been described in animal models to inhibit the growth, development, and metastasis of cancer cells, especially through the alteration of genes involved in cell cycle control and apoptosis [18]. The results of one preclinical study suggested tempe might have chemopreventive and chemotherapeutic potentials [19]. Thus, at the present time, there is clearly a great deal of interest in the development of functional foods, especially in cemented food-based medicines, because they can be prospective candidates for future anticancer functional foods. In spite of the fact that such information is available regarding the presence of different isoflavones in a variety of soy-fermented products, to the best of our knowledge, no reports have been made about the potential underlying mechanism of these fermented products for the management or treatment of cancer.

An important component of system biology is network pharmacology, which describes how biological systems, drugs, and diseases are interconnected and interact with one another in complex ways [20]. Through the analysis of huge amounts of data and identification of synergistic effects across multiple diseases, the study sheds light on the possible mechanisms of action of multifaceted bioactive substances. In addition, target-based network pharmacology has the potential to become a valuable approach to drug discovery and the development of next-generation herbal formulations or functional foods [20]. Thus, network and reverse pharmacology approaches were used in the present study for the identification of possible protein targets as well as molecular pathways modulated through isoflavones of soy-fermented foods against lung cancer, as well as to investigate the basic anticancer and anti-metastasis activities via in vitro experiments.

## 2. Materials and Methods

### 2.1. Identifying the Potential Targets of Compounds and Diseases

A SMILES code was derived from the PubChem database to obtain the predicted targets for all the isoflavone compounds found in the literature from fermented soy food, which were then entered into SwissTargetPrediction (www.swisstargetprediction.ch/, accessed on 1 February 2023) [21]. In order to find the genes that were associated with lung cancer, we used the keyword “lung cancer” as a search term and searched through the GeneCards database, which presented only genes scoring a relevance score of >30 (https://www.genecards.org/, accessed on 1 February 2023), DisGeNET (http://www.disgenet.org/, accessed on 1 February 2023) by utilizing the cutoff “score_gda > 0.1”, and OMIM (https://www.omim.org/, accessed on 1 February 2023) for the recognition of disease-associated targets.

### 2.2. Finding and Acquiring Potential Targets

The study identifies several potential targets, including those predicted for isoflavones as well as those associated with lung cancer. In order to analyze common targets, we constructed Venn diagrams using FunRich tool version 3.1.3 [22], whereas for information related to the classes of potential protein targets, we retrieved data from the Swiss target prediction database (http://www.swisstargetprediction.ch/error_page.php?error=1/search, accessed on 5 February 2023) [23].

### 2.3. Construction and Investigation of Protein–Protein Interaction Network 

The STRING database is used to study protein–protein interactions (PPIs) of selected potential targets (https://string-db.org/, accessed on 1 February 2023) [24]. The parameters for analysis were carried out with a confidence level of 0.400; based on the parameter settings, a false discovery rate (FDR) stringency of 5% was assigned to the analysis. Cytoscape was used to construct and analyze a PPI network of selected possible targets (version 3.9.1) and the results were subsequently imported into a PPI network of the potential targets with which the results were imported [25]. Based on three parameters, namely “degree”, “betweenness centrality”, and “closeness centrality”, a topological feature of the network was estimated, from which potential targets were selected.

### 2.4. Findings of Hub-Genes and GO-KEGG Pathway Enrichment Analysis

For the purpose of finding Hub genes in a network, the cytohubba plugin of the Cytoscape tool was used and the top ten hub-genes were predicted with the help of Maximal Clique Centrality (MCC) topological analysis. With the help of the DAVID database (https://david.ncifcrf.gov/, accessed on 5 February 2023), an enrichment analysis was performed to analyze the biological functions of target proteins and pathways associated with the disease [26]. A False Discovery Rate (FDR) of less than 0.05 was utilized to visualize the enriched GO terms and pathways. In order to summarize the top ten most insightful GO terms (BP, CC, and MF) using bioinformatics tools, a bubble graph was generated using SRplot (https://www.bioinformatics.com.cn/, accessed on 10 February 2023), and a top twenty KEGG pathway map was generated using ShinyGo 0.77 server (http://bioinformatics.sdstate.edu/go/, accessed on 1 February 2023).

### 2.5. Molecular Docking Analysis

The interaction between fermented soy products’ isoflavones and lung cancer targets was investigated using AutoDock Vina 1.5.7 [27]. From the PubChem database, the 3D structures of isoflavones were downloaded. The 3D structures of each compound were converted from .sdf to .pdb using Open Babel 3.1.1. Avogadro was used to minimize energy using the MMFF94 force field. The steepest descent algorithm was used to optimize the model, and a total of 5000 steps were taken in order to optimize it. In order to minimize the energy, the structure was updated continuously, and when the energy difference was less than 0.1, the minimization was terminated and then the .PDB file was saved. Protein 3D crystal structures were downloaded from RCSB-PDB database (TNF—PDB ID: 2AZ5, SRC—PDB ID: 4MXO, MMP9—PDB ID: 1GKC, ANXA5—PDB ID: 1AVH, CASP3—PDB ID: 1NME, HRAS—PDB ID: 6ZL3, PTGS2 PDB ID—5F1A, TP53—PDB ID: 3DCY, HSP90AA1—PDB ID: 5H22 and ALB—PDB ID: 1AO6). Water molecules were deleted from the crystal structure. The protein structure was then charged with Kollman charge and hydrogen was added. The coordinates of the proteins were saved as .pdb file. All structures were adapted using Open Babel, from .pdb to .pdbqt. An analysis of docked protein-ligand complexes was conducted using PyMoLv2.5.5 and Biovia Discovery Studio v-21.1.0.20298 [28].

### 2.6. ADMET Prediction

After molecular docking analysis, isoflavones were screened according to their ADMET properties. Using the Protox-II server (https://tox-new.charite.de/protox_II/index.php?site=home, accessed on 25 January 2023) [29] and SwissADME (http://www.swissadme.ch/, accessed on 25 January 2023) [30], ADMET properties as well as patterns of PAINS (pan-assay interference compounds) were predicted. PAINS patterns were filtered out after selecting compounds with good ADMET properties [31]. Using the PAINS filter, we could eliminate compounds that have specific patterns and a high propensity to bind to more than one target. ADMET evaluates compounds for their drug-like physicochemical and pharmacokinetic properties, which decrease the possibility of clinical trial failure [32].

### 2.7. Molecular Dynamics Simulation

Computational methods such as molecular dynamics (MDs) are used to understand how ligands behave in the binding pocket of receptors based on their time-dependent conformational stability. Several studies have demonstrated its practical application in identifying new inhibitors in a number of applications [33,34,35,36]. In this study, MD analyses were performed with Gromacs version 2019.4 [37]. MD studies were conducted using the GROMOS force field. For obtaining the force field coordinates, the chosen ligand topology was retrieved from the ATB server. Using the steepest descent algorithm, 1500 steps of vacuum minimization were performed on the system. Using a simple point charge (SPC) water model, the complex structures were solvated using a cubic periodic box of 0.5 nm. Complex systems were maintained at an appropriate salt concentration of 0.15 M by adding appropriate numbers of Na^+^ and Cl^−^ counterions. The NVT and NPT equilibration was performed for 100 ps steps using the leap-frog algorithm. After equilibration, the solvated protein–ligand complex was subjected to the production of MDs for 100 ns. A trajectory file was further analyzed after the periodic boundary conditions were removed from the MD run. Using the Chimera package, the MD analysis was carried out on the data. Diagrams were generated using the XMGRACE tool (https://plasma-gate.weizmann.ac.il/Grace/, accessed on 5 February 2023).

### 2.8. Pass Analysis

The concept of PASS refers to a method of predicting the properties of biologically active compounds based on their chemical structure, which makes it possible to predict chemical compounds’ biological activities. Using the PASS web server (https://www.way2drug.com/passonline/, accessed on 25 January 2023), it is possible to perform PASS analysis on compounds and select those with the desired biological properties [38]. According to structure–activity relationships, PASS predicts a compound’s potential biological properties. Accordingly, it determines a compound’s probable properties on the basis of a measure of Pa to Pi (probability of being active to probability of being inactive). In general, compounds with higher Pa values are more likely to be active. For higher statistical significance, the Pa value was set at >7.

### 2.9. Cell Culture

A-549 lung cancer cells were cultured in Dulbecco’s Modified Eagle Medium (DMEM), containing 10% FBS (fetal bovine serum), 10,000 units of penicillin with 5 mg of streptomycin (Hi-Media^®^, Mumbai, India). Cells were maintained at 37 °C in a humidified atmosphere containing 5% CO_2_ to maintain their optimum state [38].

### 2.10. Cell Viability Assay

Using a MTT assay, lung cancer cells treated with daidzein were evaluated for viability. By trypsinizing and aspirating cells from T-25 flasks, cells were harvested. Centrifugation at 3000× *g* rpm was used to collect the cells. In order to adjust the cell count, a culture medium was used to suspend around 10,000 cells in 200 µL. To settle the cells, a 200 µL suspension of cells was placed in every well of a 96-well microtiter plate and incubated at 37 °C in 5% CO_2_ for 24 h. At the end of 24 h of incubation under 5% CO_2_ conditions at 37 °C, cells were treated with 200 µL of various concentrations of standard daidzein after the removal of spent medium from each well (Sigma-Aldrich^®^, St. Louis, MO, USA) (0, 1, 10, 100, 250, 500, and 1000 µg/mL), followed by further incubation of 24 h under 5% CO_2_ at 37 °C. In the next step, 200 µL of freshly prepared medium was poured into each well along with 10% MTT reagent. It was further incubated under 5% CO_2_ for 3 h at 37 °C. As the formazan crystals developed, DMSO (100 µL) was added and gently shook in a gyratory shaker to dissolve them. In order to determine absorbance at 570 nm and 630 nm, a microplate reader was used. After subtracting the background and blank values from the results, the amount of drug required to inhibit 50% of the cell growth (IC50) was determined [39,40].

### 2.11. Wound-Healing Assay

A wound-healing assay was used to study the effects of daidzein on A549 cancer cells. In 6-well plates, monolayers of cells were grown. A density of 1 × 10^6^ cells/mL in 3 mL of medium was used for plating. A sterile 1 mL pipette tip was used to make an injury line in the middle of the culture. Each scraped line was photographed using an inverted microscope. Later on, daidzein was added to the wells at different concentrations (50, 80, and 100 µg/mL) and incubated for another 48 h before images were taken. A reduction in the scraped area indicates the migration of cells and healing of wounds [41].

### 2.12. Transwell Migration Assay

Transwell inserts (24-well format, 8 m pore size, Himedia^®^, Mumbai, India) were utilized for seeding 1 × 10^6^ cells in media (serum-free). The cells were then cultured for 24 h with various concentrations of daidzein (50, 80 and, 100 µg/mL). A 10% FBS solution was added to the lower chamber. Following incubation for 10 h, non-moving cells were removed using a cotton swab. The migrated cells were fixed with methanol and stained using crystal violet (0.1%). Under an inverted microscope, cell numbers were also counted by calculating the mean of three randomly selected fields. A calculation was then made to determine how many cells had penetrated the membrane [42]. 

## 3. Results

### 3.1. Target Prediction and Analysis of Potential Targets

**Figure 1:**
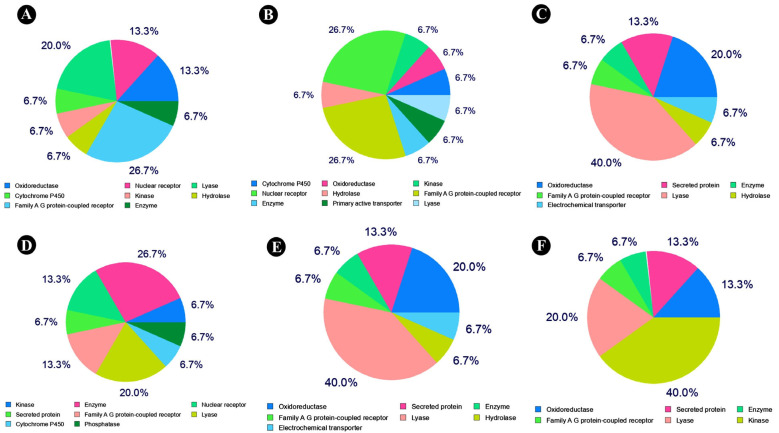
The protein classification of targets of isoflavones in soy-fermented food products against lung cancer, as retrieved from the SwissTarget-Prediction server: (**A**) Daidzein, (**B**) genistein, (**C**) genistin, (**D**) glycitein, (**E**) malonyldaidzin, and (**F**) malonylgenistin.

**Figure 2:**
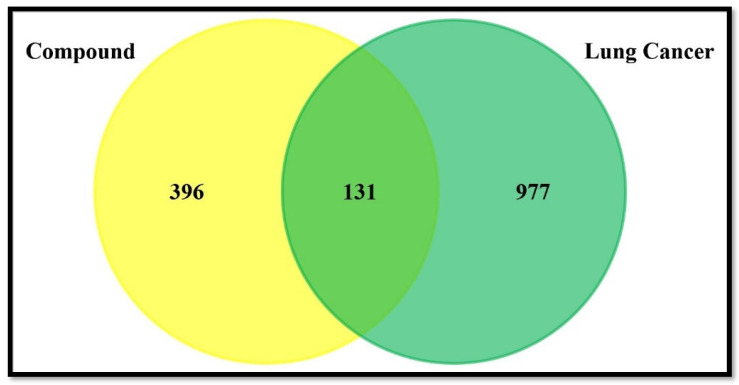
Findings of common targets between isoflavones and lung cancer using a Venn diagram.

As a result of the literature search, six major isoflavones were identified from the different soy-fermented foods that have been incorporated into this study (Table 1). From the PubChem database, detailed information about the selected compounds was retrieved and analyzed using the SwissTargetPrediction (STP) database. The target classes of each isoflavone are shown in Figure 1. In total, 527 predicted targets were obtained from the Swisstarget prediction and PharmMapper webserver, while 1108 target predictions were obtained from the Genecards (GDA cutoff of >30), DisGeNet (cutoff of >0.1), and OMIM databases after removing duplicate targets. In the process of intersecting the targets of compounds and diseases, a total of 131 common targets were found to be potential targets (Figure 2).

### 3.2. Construction and Analysis of Compounds–Disease Common Target Network

**Figure 3:**
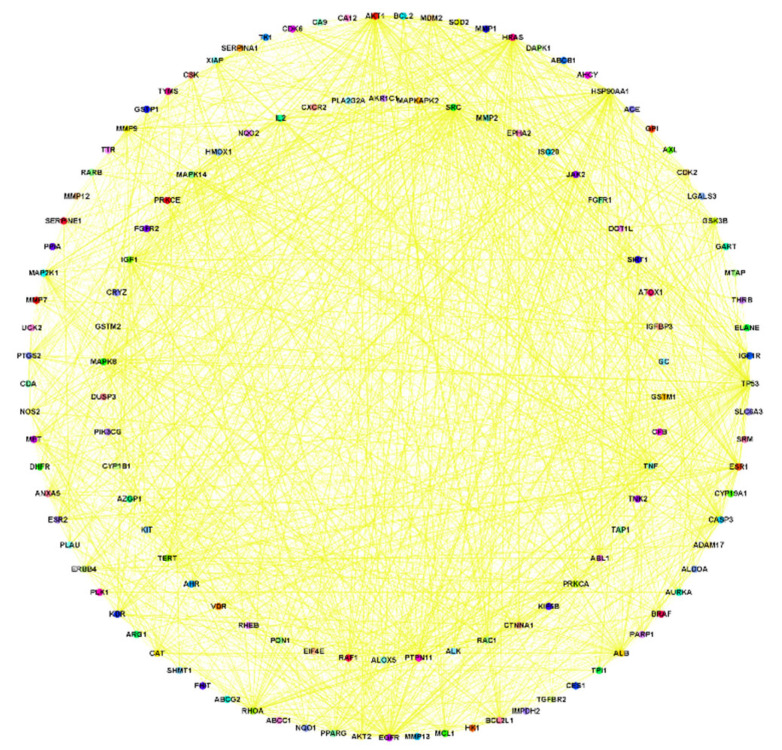
The network of common-gene targets for lung cancer and isoflavones in soy-fermented food products created with Cytoscape software.

**Figure 4:**
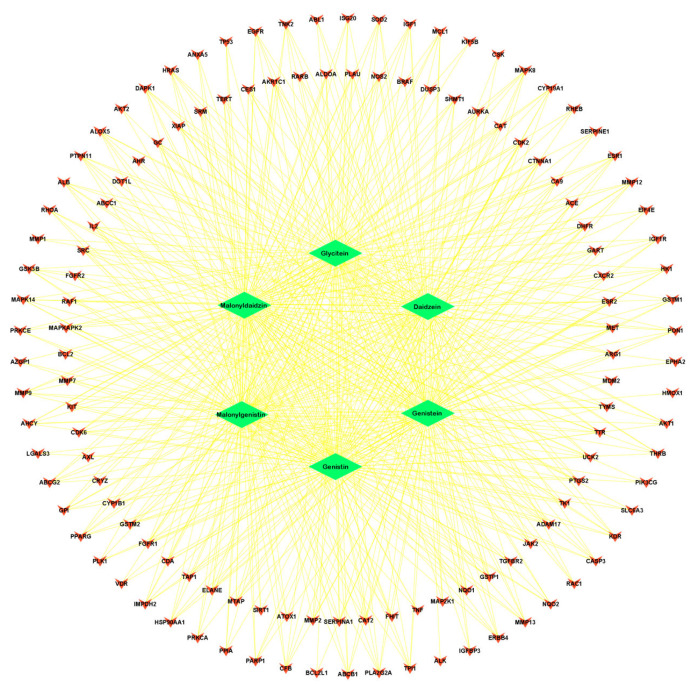
The common-gene targets for isoflavones of soy-fermented food products (green diamonds) and their associations (yellow edges) with the genes involved in lung cancer (pink ‘V’ shape), visualized using Cytoscape software.

An analysis of the relationship between target genes was conducted using a PPI network. The first step in this process was the entering of possible targets using the STRING database, and then, once the data were collected, we used Cytoscape version 3.9.1 to analyze and visualize the resultant data (Figure 3 and Figure 4). 

**Figure 5:**
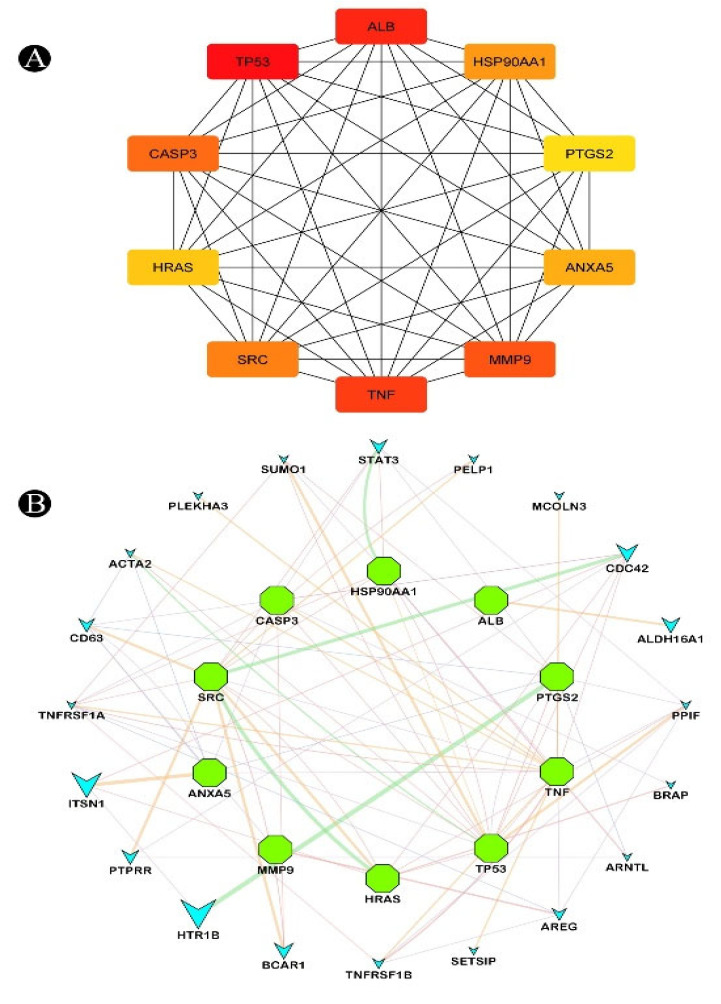
(**A**) Identified hub genes in a PPI network obtained from common target genes of isoflavones in soy-fermented food products and lung cancer; (**B**) network of hub genes against lung cancer analyzed by GeneMANIA. Functional association of targets was analyzed and connecting lines with different colours represent different correlations. Genes associated with query genes were indicated by nodes on the outer ring. The genes shown in the inner ring were used as search terms to find relevant information.

For the estimation of the significance of different nodes within a complex network, three parameters were used: degree, closeness, and centrality between the nodes (Table 2 and Table 3). Each of these three parameters were used in order to estimate the importance of each node in relation to the rest of the network. The genes that were identified in the study have been reported to play a significant role in lung cancer development. In this regard, these findings indicate that the anticancer activity exhibited by the isoflavones in soy-fermented foods can be significantly associated with these key targets. Considering the topology properties of the network, we found ten targets in the network, corresponding to TP53, ALB, TNF, MMP9, CASP3, SRC, HSP90AA1, ANXA5, HRAS, and PTGS2, which were arranged in the order of high to low (Figure 5A). These ten identified targets could serve as the key targets that isoflavones can target in order to prevent lung cancer. Additionally, we used the GeneMANIA tool to export the identified target genes into a PPI network so we can see what kind of relationships there might be between the identified target genes as well as other additional genes present in the network. According to the results, the interactions in the network represents the weight and expressed as percentages. According to the analysis of all interactions between targets in the network, it was estimated that 23.39% of the interactions involved co-expressions and 18.91% of them involved physical interactions between the targets. The results of the study also revealed that there was a correlation between genetic interactions (28.43%), predicted interactions (26.42), and colocalization (2.85%) (Figure 5B).

### 3.3. Functional and Pathway Enrichment Analysis

**Figure 6:**
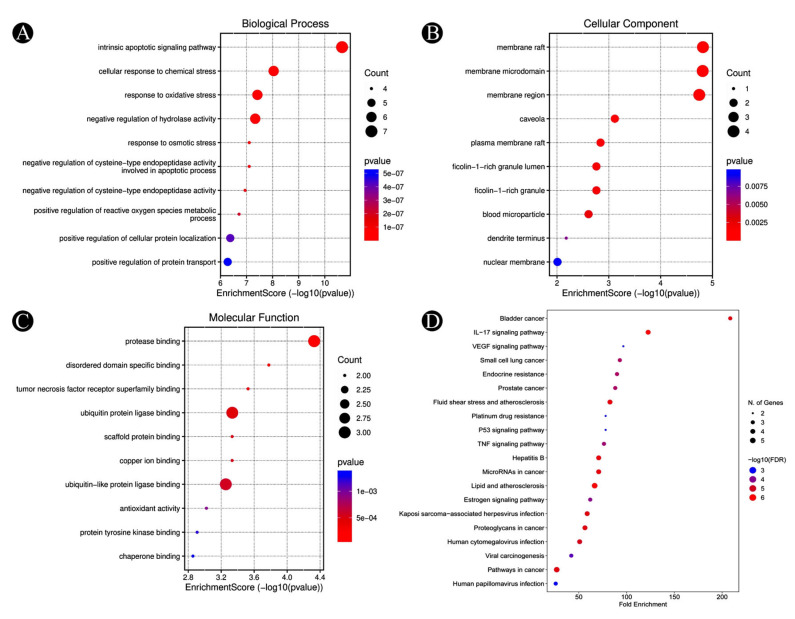
KEGG pathway and GO enrichment analyses of identified hub-target proteins (*p*-value ≤ 0.05). (**A**) The biological processes (top 10), (**B**) the cellular components (top 10), (**C**) the molecular functions (top 10), (**D**) the KEGG pathways (top 20). The *p*-values for each term are shown by the colours, with darker colours indicating lower *p*-values. The number of genes associated with each term are indicated by the dot sizes, with larger dots representing more genes.

With the help of Enrichr, the identified target genes in lung cancer were analyzed to clarify their functions and pathways for an in-depth understanding of how they relate to the disease process, thus allowing us to better grasp the disease process in general. As a result of the GO annotations, a variety of GO enrichment terms have been found to be associated with the annotations. A total of 1577 BPs, 75 CCs, and 119 MFs were determined. An analysis of this data was performed using a bubble chart, which represents the ten most enhanced GO functions (Figure 6A–C) and each KEGG pathway (Figure 6D). These identified genes were found to play a role in biological processes such as the intrinsic apoptotic signaling pathway, response to osmotic stress, positive regulation of cellular protein localization, cellular response to chemical stress, response to oxidative stress, regulation of the apoptotic signaling pathway, and control of the neuroinflammatory response. They also played a role in cellular components, such as the membrane microdomain, membrane raft, caveola, plasma membrane raft, ficolin-1-rich granule, nuclear membrane, and dendrite terminus, and in molecular functions such as protease binding, tumor necrosis factor receptor superfamily binding, ubiquitin protein ligase binding, copper ion binding, heme binding, chaperone binding, antioxidant activity, and protein tyrosine kinase binding. There were 163 KEGG pathways that have been linked to these genes. Among them, small-cell lung cancer, the IL-17 signaling pathway, pathways in cancer, prostate cancer, the P53 signaling pathway, the TNF signaling pathway, the VEGF signaling pathway, and the estrogen signaling pathway were the most significantly enriched pathways. From the obtained results, it can be predicted that soy-fermented food compounds can regulate some signaling pathways in lung cancer.

### 3.4. Molecular Docking and ADMET Analysis

**Figure 7:**
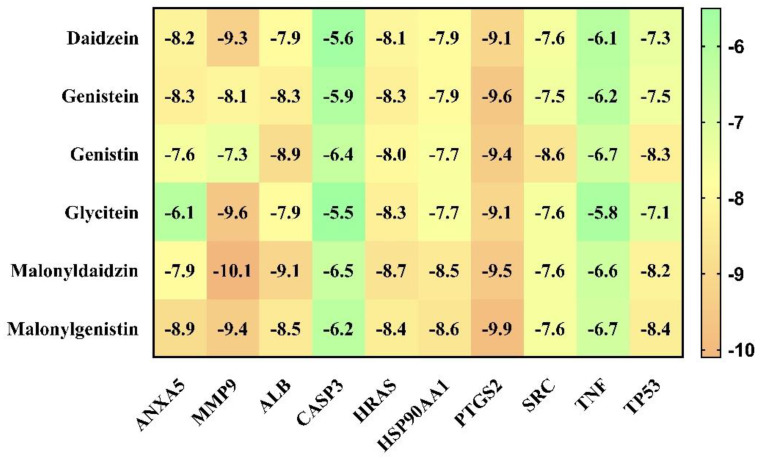
Binding energy of the top-rated pose of a ligand–receptor complex obtained after molecular docking analysis.

The results of the molecular docking analysis of isoflavones against the identified lung cancer targets are presented in Figure 7. Structures with lower binding energies are generally more stable. Several isoflavones, such as Malonylgenistin against ANXA5, HSP90AA1, PTGS2, TNF, and TP53; Malonyldaidzin against MMP9, ALB, CASP3, and HRAS; and Genistin against SRC, were found to have a higher affinity for their respective target proteins when docking analysis was performed. 

**Figure 8:**
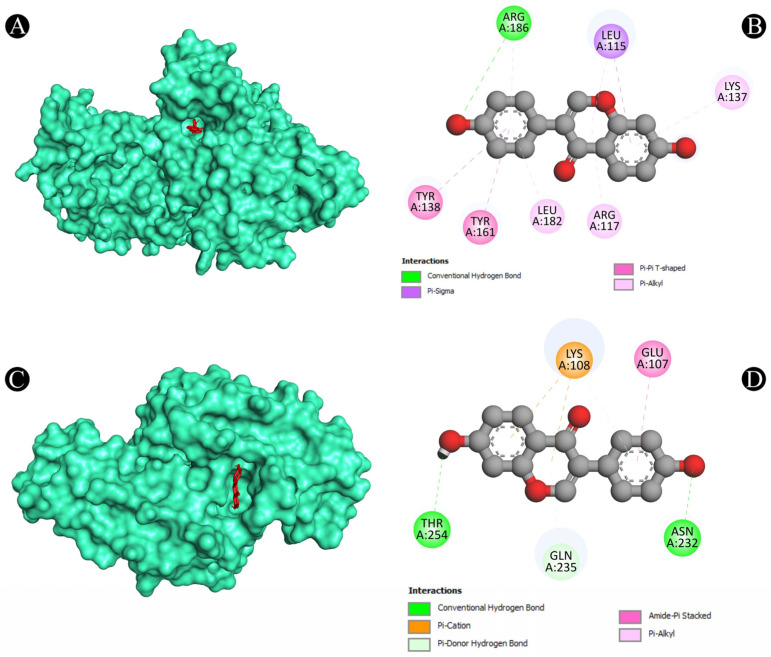
(**A**,**B**) The result of the molecular docking study showing how daidzein binds to ALB, (**C**,**D**) the result of the molecular docking study showing how daidzein binds to ANXA5 protein.

**Figure 9:**
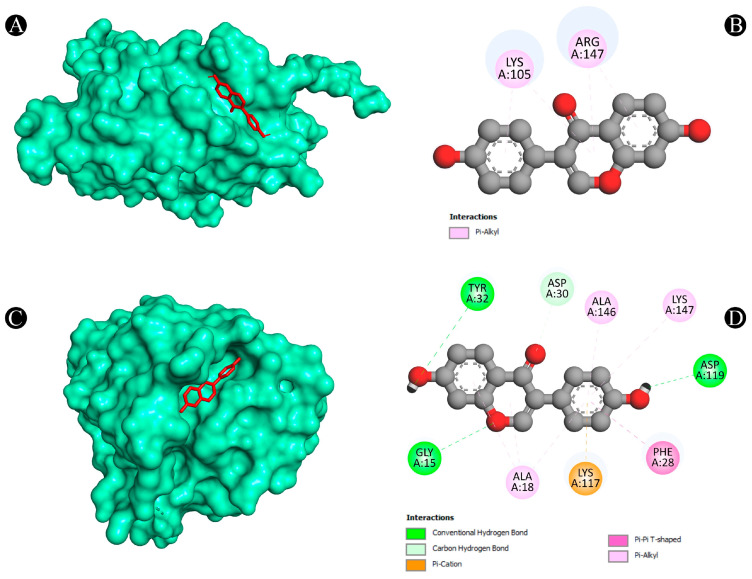
(**A**,**B**) The result of the molecular docking study showing how daidzein binds to CASP3, (**C**,**D**) the result of the molecular docking study showing how daidzein binds to HRAS.

**Figure 10:**
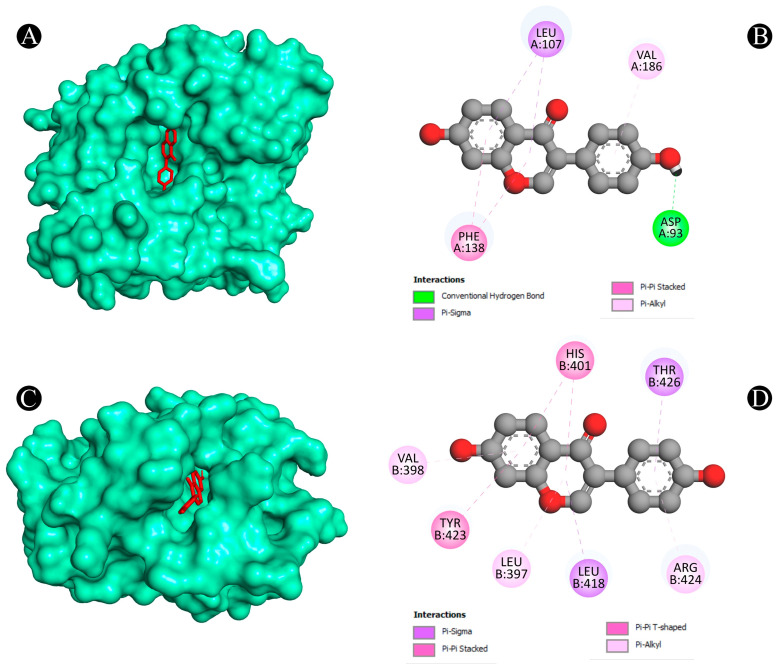
(**A**,**B**) The result of the molecular docking study showing how daidzein binds to HSP90AA1, (**C**,**D**) the result of the molecular docking study showing how daidzein binds to MMP9.

**Figure 11:**
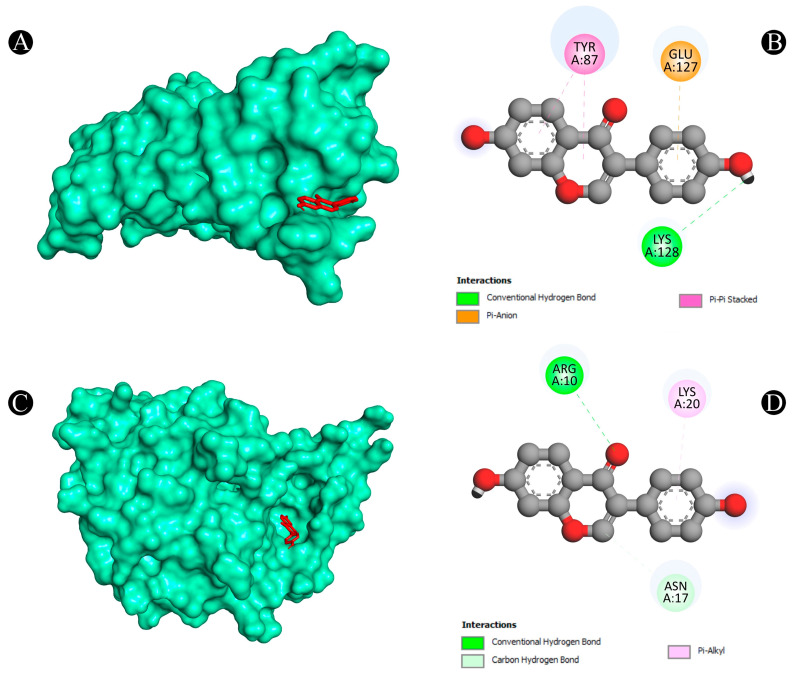
(**A**,**B**) The result of the molecular docking study showing how daidzein binds to TNF, (**C**,**D**) the result of the molecular docking study showing how daidzein binds to TP53.

**Figure 12:**
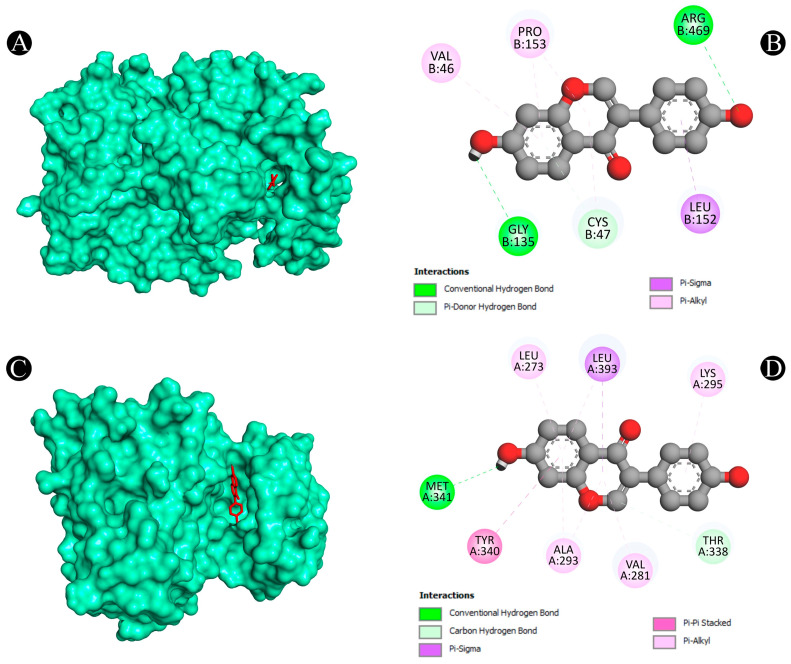
(**A**,**B**) The result of the molecular docking study showing how daidzein binds to PTGS2, (**C**,**D**) the result of the molecular docking study showing how daidzein binds to SRC.

Furthermore, the Protox-II and SwissADME servers were used to detect the ADMET properties and PAINS patterns of isoflavones. A list of ADMET properties is presented in Table 4 and Table 5. As a result of the analysis, only daidzein showed good ADMET properties and did not show any PAINS patterns as a result. Aside from daidzein, other isoflavones were excluded from further evaluation, although a few have good binding affinities with lung cancer target proteins. The results of daidzein showed good binding energy towards MMP9 (−9.3 kcal/mol), with two pi–sigma bonds (LEU418 and THR426), two pi–pi stacked bonds (2*HIS401), one pi–pi t-shaped bond (TYR423), and three pi–alkyl bonds (LEU397, VAL398, and ARG424); towards PTGS2 (−9.1 kcal/mol), with two conventional H-bonds (ARG469 and GLY135), one pi–donor hydrogen bond (CYS47), one pi–sigma bond (LEU152) and five pi–alkyl bonds (2*CYS47, 2*PRO153, and VAL146); towards ANXA5 (−8.2 kcal/mol), with two conventional H-bonds (ASN232 and THR254), two pi–cation bonds (2*LYS108), two pi–donor hydrogen bonds (LYS108 and GLN235), one amide–pi stacked bond (GLU107) and two pi–alkyl bonds (2*LYS108); towards HRAS (−8.1 kcal/mol), with three conventional H-bonds (GLY15, ASP119, and TYR32), one carbon–hydrogen bond (ASP30), one pi–pi t-shaped bond (PHE28), and six pi–alkyl bonds (3*ALA18, LYS117, ALA146, and LYS147); towards HSP90AA1 (−7.9 kcal/mol), with one conventional H-bond (ASP93), two pi–sigma bonds (2*LEU107), two pi–pi stacked bonds (2*PHE138), and one pi–alkyl bond (VAL186); towards ALB (−7.9 kcal/mol), with one conventional H-bond (ARG186), one pi–sigma bond (LEU115), one pi–pi t-shaped bond (TYR138 and TYR161), and five pi–alkyl bonds (LEU115, ARG117, LYS137, LEU182, and ARG186); towards SRC (−7.6 kcal/mol), with one conventional H-bond (MET341), one carbon–hydrogen bond (THR338), one pi–sigma bond (9LEU393), one pi–pi stacked bond (TYR340), and six pi–alkyl bonds (VAL281, 2*ALA293, LEU273, LEU393, and LYS295); towards TP53 (−7.3 kcal/mol), with one conventional H-bond (ARG10), one carbon–hydrogen bond (ASN17), and one pi–alkyl bond (LYS20); towards TNF (−6.1 kcal/mol), with one conventional H-bond (LYS128), one pi–anion bond, (GLU127) and two pi–pi stacked bonds (2*TYR87); and towards CASP3 (−5.6 kcal/mol), showing four pi–alkyl bonds (2*LYS105 and 2*ARG147). The interaction analyses of daidzein with target proteins are presented within in Figure 8, Figure 9, Figure 10, Figure 11 and Figure 12 as well as in Table 6.

### 3.5. MD Simulation Analysis

**Figure 13:**
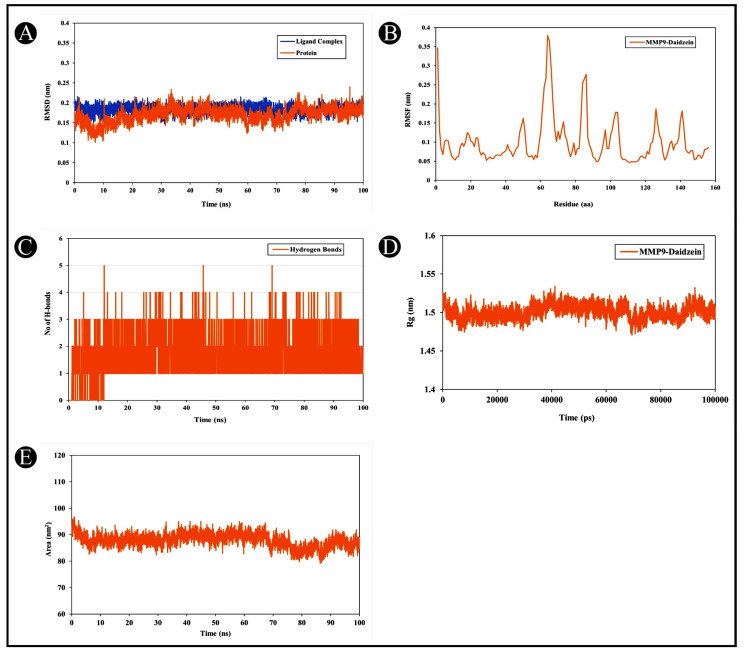
The molecular dynamics simulation analysis of MMP9 protein and daidzein molecule over time. Molecular dynamics of MMP9 and its binding with diadzein. (**A**) RMSD analysis of MMP9 with and without diadzein binding, (**B**) RMSF analysis of MMP9–diadzein complex, (**C**) MMP9–diadzein complex intermolecular H-bond time evolution within 0.35 nm, (**D**) the Rg distribution of MMP9–diadzein complex, (**E**) SASA plot analysis of MMP9–diadzein complex.

To understand the protein–ligand stability as well as the protein structural flexibility between the docked complex of daidzein–MMP9, further MD simulation using GROMACS software was performed at 100 ns. Proteins and protein–ligand complexes can be examined using RMSD analysis to determine structural deviations. During the simulation, the structural deviations of MMP9 and the MMP9–daidzein complexes were investigated in the solvent environment to determine their stability and movement. As a result of the simulation, the RMSD values of the backbone of MMP-9 and the docked complex by daidzein showed a stable pattern (Figure 13A). MMP9 and the MMP9–daidzein complex showed an average RMSD of 0.17 nm and 0.18 nm, respectively, leading to a maximum RMSD of 0.21 nm at certain points. As a result of the initial adjustments, random fluctuations in the RMSD pattern were seen in both MMP9 systems after 10 ns. Throughout the simulation, there were no significant shifts in the RMSD pattern, suggesting that MMP9 was stable despite a strong ligand-binding strength. The RMSF of a protein is a measure of the flexibility of every residue within it. There was an average fluctuation of 0.10 nm in MMP9–daidzein complex during the simulation. Following daidzein binding, the fluctuations appeared stable and minimized. Based on the graph, it appears that MMP9 and daidzein interact with remarkable constancy (Figure 13B). The protein structures depend on H-bonds for stability and integrity. As a means of assessing the integrity and stability of docked protein–ligand complexes, the MMP9–daidzein complex’s intermolecular hydrogen bonds promote both protein and ligand stability. The MMP9–daidzein-docked complex was maintained by three H-bonds. A simulation was therefore carried out in order to examine their time evolution during the simulation process (Figure 13C). According to the plot, MMP9 and daidzein formed an average of three H-bonds, which were quite stable during simulation. Daidzein formed five H-bonds at different locations, but the number remained the same (three). Molecular stability can also be calculated from the compactness of protein molecules. The compactness measure in MD simulations is called Rg. The compactness of a protein structure is a useful parameter that can be used to examine the tertiary structure. Rg values were used to assess the compactness of MMP9 after daidzein binding. According to Figure 13, the MMP9–daidzein complex had an average Rg value of 1.50 nm (Figure 13D). In the Rg plot, the protein–ligand complex remained compact during the simulation. Basically, SASA refers to how much surface area is accessible to a neighboring solvent from the surface of a protein molecule. During simulations, SASA analysis is widely used to examine protein folding or unfolding and structural stability. Based on the simulation, there were no major peaks in SASA values, indicating that daidzein binding affected MMP9 folding behaviour. For the MMP9–daidzein complex, the average SASA value was 87.88 nm^2^. The SASA values showed that MMP9 remained stable in the presence of daidzein (Figure 13E).

### 3.6. PASS Analysis of Daidzein

In order to identify safe and effective lead molecules for drug discovery and development, it is essential to assess the biological properties of the compound under investigation. To explore the biological properties of daidzein, a PASS analysis was performed. The results showed that daidzein was found to be an aldehyde oxidase inhibitor, histidine kinase inhibitor, HIF1A expression inhibitor, membrane integrity agonist, MMP9 expression inhibitor, antimutagenic, membrane permeability inhibitor, AR expression inhibitor, TP53 expression enhancer, RELA expression inhibitor, apoptosis agonist, JAK2 expression inhibitor, and HMOX1 expression enhancer, with significant Pa values ranges from 0.712 to 0.967 (Table 7). 

### 3.7. Anticancer Activity of Daidzein

**Figure 14:**
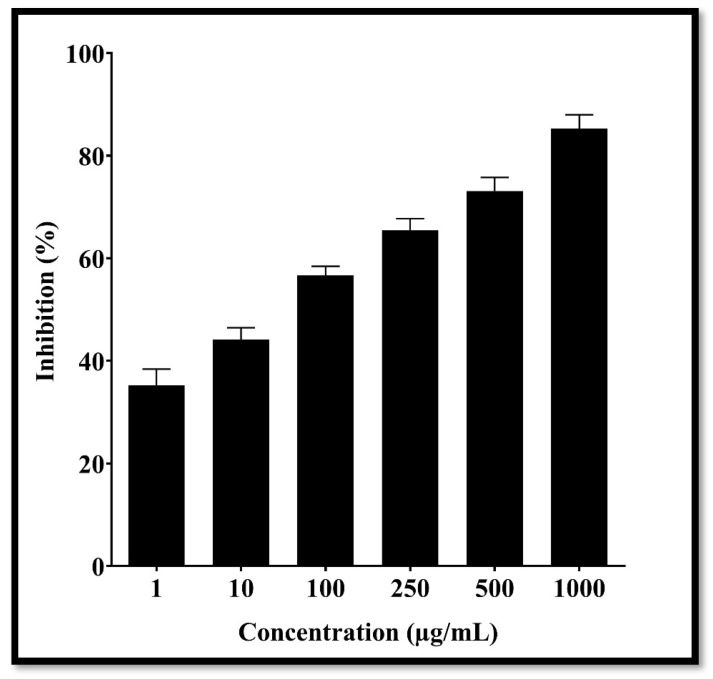
MTT assay in A549 cells with and without daidzein at different concentration for 24 h. Each value represents the mean of three independent experiments conducted in triplicate.

The anticancer activity of standard daidzein was checked via MTT assay against A549 lung cancer cells. Based on the obtained results, lung cancer cell viability was inhibited in a time- and dose-dependent manner in response to the treatment at various concentrations of daidzein. It was found that daidzein had an IC50 value of 83.06 µg/mL against A549 lung cancer cells (Figure 14). 

### 3.8. Anti-Metastasis Activity of Daidzein

**Figure 15:**
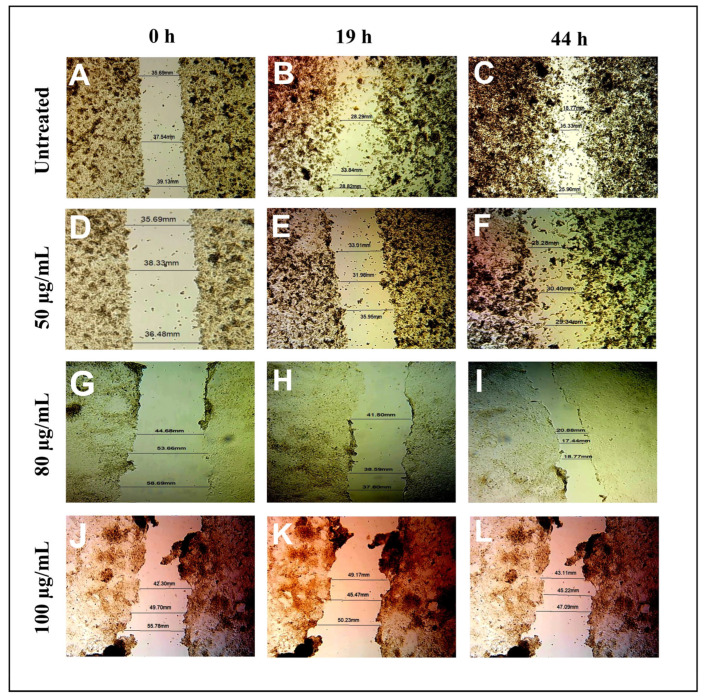
The effect of diadzein on the migration of A549 cells. (**A**–**C**) At 0, 19, and 44 h, a scratch was made in a monolayer of untreated A549 cells, (**D**–**F**) after treatment of a 50 µg/mL concentration of diadzein at 0, 19, and 44 h, (**G**–**I**) after treatment with a 80 µg/mL concentration of diadzein at 0, 19, and 44 h, (**J**–**L**) after treatment with a 100 µg/mL concentration of diadzein at 0, 19, and 44 h.

**Figure 16:**
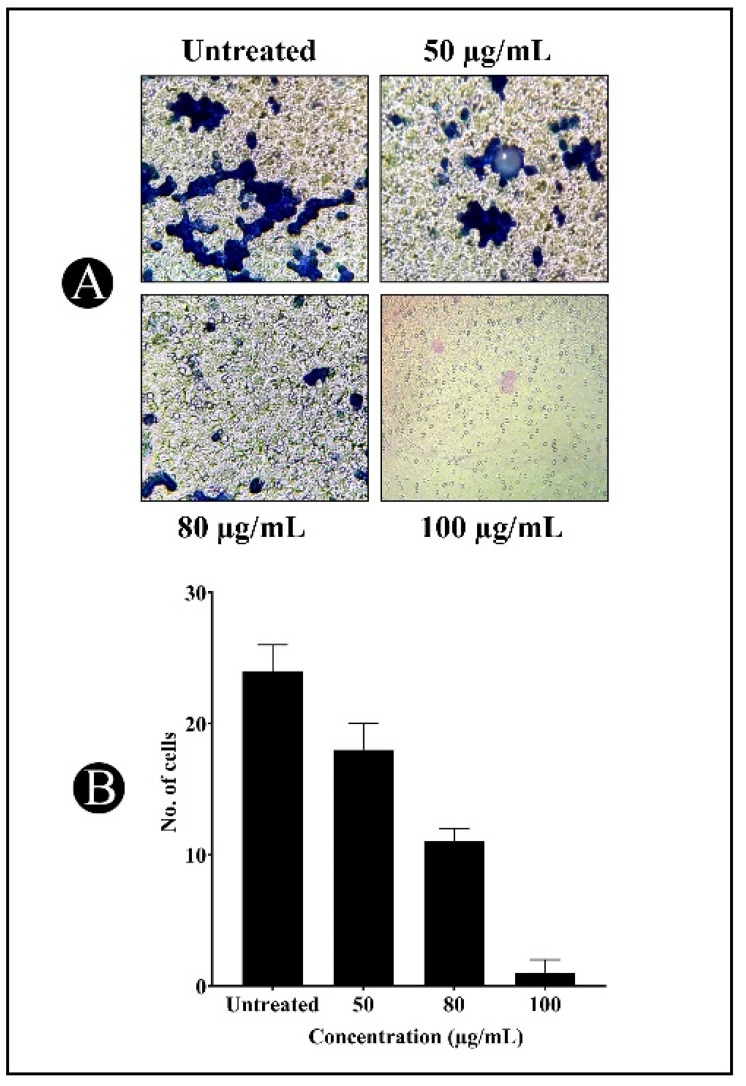
(**A**) Using Transwell^®^ cell culture chambers, migration assay of A549 cells treated with different concentrations of diadzein for 24 h; (**B**) at random, five regions of each well are represented by bar graphs (*n* = 3).

As a result of the progression of cancer in several epithelial cells, cell migration is usually considered the most significant metastatic event taking place during cancer progression. Consequently, wound healing and a transwell invasion assay were performed to investigate if daidzein inhibited A549 cancer cell migration. The outcomes of the experiments shown in Figure 15 show that untreated cancer cells from A549 slowly moved from the wound site to the clear area after 19 and 44 h of wounding. Different concentrations of daidzein inhibited the migration of the A549 cancer cells. The A549 cancer cells were inhibited in a dose-dependent manner by daidzein treatment in the transwell invasion assay, as illustrated in Figure 16.

## 4. Discussion

The field of network pharmacology has emerged as an area that combines various aspects of systems biology, bioinformatics, and network science to analyze molecular interactions between drugs and the targets of their treatment. There is a growing interest in revealing the systematic pharmacological mechanisms of drugs, which can help guide research and development as well as the clinical diagnosis and treatment of new drugs and drugs in the pipeline [43]. This approach has been successful in elucidating many complex and complicated therapeutic mechanisms of herbal and functional foods [44,45,46]. Various types of fermented soy products have been investigated for their possible anticancer properties due to their unique composition and fermentation process. Isoflavones are a class of phytoestrogens that are considered to be one of the primary bioactive compounds in these products. These compounds have been examined in several studies in order to determine their impact on different types of cancerous cells [18,19]. Despite this, there are only a few scientific studies that provide evidence supporting the therapeutic mechanism of isoflavones found in soy-fermented food products against lung cancer. Therefore, in this study, we investigated the potential active components and their possible mechanisms of isoflavones found in fermented soy products in terms of treating lung cancer using network pharmacology. The results of the analysis were validated by conducting functional assays in vitro and looking at the potential anticancer effect as well as anti-metastasis effect.

A protein–protein interaction (PPI) network is a powerful tool in various fields of biological and biomedical research. PPI networks are graphical representations that depict interactions between proteins within a cell or organism. These networks are valuable for understanding the complexity of cellular processes, signaling pathways, disease mechanisms, and drug discovery [47]. When a protein becomes abnormal, it can lead to various biological consequences and may contribute to the growth of diseases including cancer [48]. In this study, TP53, ALB, TNF, MMP9, CASP3, SRC, HSP90AA1, ANXA5, HRAS, and PTGS2 were identified as the top ten targets in the PPI network constructed. TP53 is also known as p53, and cells are protected from becoming cancerous by this tumor suppressor gene. Mutations of this gene are prevalent in various cancers, including lung cancer. TP53 is located on chromosome 17 and encodes the p53 protein, responsible for regulating multiple target genes, especially those involved in cell cycle control, DNA repair, apoptosis (programmed cell death), and senescence (cellular aging) [49]. ALB (serum albumin) is an important indicator for monitoring the nutritional status of cancer patients. Lower levels of Alb are associated with a worse prognosis for patients with malignant tumors [50]. The tumor necrosis factor (TNF) is a multifunctional cytokine that plays a variety of roles in cancer, including lung cancer. TNF helps in regulating inflammation, immunity, cell survival, and apoptosis [51]. In many biological processes, matrix metalloproteinase-9 (MMP-9) plays a vital role. Numerous studies have linked it to cancer pathology, including, but not limited to, invasion, angiogenesis, and metastasis [52]. Early in tumorigenesis, it contributes to the creation of the metastatic niche and promotes the colonization of the lungs by circulating tumor cells [53]. Lung cancer malignancy is positively correlated with its expression [54]. Caspase-3 (CASP3) is crucial in tumorigenesis and cancer progression as a key enzyme in the apoptotic pathway. Researchers often use CASP3 activation as a substitute marker for assessing cancer treatments’ effectiveness [55,56]. Cell survival and tumorigenesis in non-small cell lung cancer (NSCLC) are maintained by SRC protein interactions with cell surface growth factor receptors and intracellular pathways [57]. Many oncologic functions are mediated by SRC-family kinases in cancers, including migration, proliferation, survival, motility, and angiogenesis [58]. HSP90AA1 is a gene that encodes the protein HSP90. Multi-client proteins of this chaperone protein play a key role in cancer development. A study found a correlation between poorer overall survival and higher expression of HSP90AA1 in cancer tissues. The expression and transcription of HSP90AA1 as well as the activity of the AKT1/ERK pathways were found to be higher in the tissues of lung cancer patients [59]. According to another study, CDK1 and HSP90AA1 were also found to be common in their analysis, suggesting that CDK1 and HSP90AA1 play an important role in the regulation of non-small cell lung cancer [60]. Annexin A5 is encoded by the ANXA5 gene. As phospholipid-binding, calcium-regulated protein, it regulates cell cycle, exocytosis, and apoptosis. By regulating the expression of Bcl-2 and Bax when ANXA5 expression was increased, cell proliferation was inhibited, whereas cell metastasis was suppressed by regulating E-cadherin and MMP-9 expression [61]. In the RAS family of proto-oncogenes, HRAS is a gene that encodes for the protein HRAS. The genes encode proteins that regulate the growth, differentiation, and survival of many types of cells [62]. There is a gene called PTGS2 that encodes a protein called Prostaglandin-endoperoxide synthase 2 (PTGS2), also referred to as cyclooxygenase-2 (COX-2). The PTGS2 gene plays a role in a number of pathological processes, including non-small cell lung cancer chemoresistance [63]. 

The results of the KEGG enrichment analysis showed that the estrogen signaling pathways, TNF signaling pathways, P53 signaling pathways, VEGF signaling pathways, and IL-17 signaling pathways were significantly enhanced in this study. There is evidence that the estrogen signaling pathway contributes to the development of lung cancer. By activating certain signaling pathways, estrogen can up-regulate the expression of certain genes and promote lung cancer cell migration. Additionally, estrogen has the ability to transactivate growth factor signaling pathways, specifically the epidermal growth factor pathway [64]. There is evidence to suggest that the VEGF signaling pathway contributes to the development of lung cancer. There is no doubt that VEGF plays an important role in angiogenesis, a process that is essential for tumor growth and propagation. It is important to recognize that VEGF plays a pivotal role in the establishment of a vascular supply within a tumor, which plays a vital role in the progression of lung cancer. Consequently, a new class of drugs has emerged, aimed at inhibiting its pathway, and its efficacy has been demonstrated in improving patient prognoses [65]. As a major mediator of inflammation-induced cancer, TNF plays an important role. A rapid rise in the level of TNF has been found to be a universal adaptive response to the inhibition of EGFR in NSCLC, regardless of whether the EGFR is expressed or not. The EGFR signaling inhibits the production of TNF mRNA by inducing miR-21 expression, which in turn has the effect of decreasing the stability of the TNF mRNA [66]. The p53 protein plays a crucial role in maintaining the integrity of the genome, which is why it is referred to as the “guardian of the genome”. Approximately half of all cancers are caused by mutations in the TP53 gene, including those of the lung, breast, colon, prostate, liver, bladder, and skin. DNA damage stops the cell cycle when the TP53 gene on human chromosome 17 is activated. An unrestricted cell cycle and an uncontrolled reproduction of damaged DNA result from mutations in the p53 protein, leading to cancer tumors. [67,68]. Inflammatory processes are facilitated by IL-17, a cytokine that plays a crucial role in cancer development. By activating the IL-6-Stat3 signaling pathway, IL-17 can accelerate tumor growth. When IL-17 levels rise, IL-6 production increases, which activates the oncogenic signal transducer and activator of transcription (STAT) 3, resulting in prosurvival and proangiogenic genes being expressed. As a result, IL-6–Stat3 plays a role in promoting tumor growth through the Th17 response [69,70,71]. There are several inhibitors which are reported to inhibit the TNF, p53, VEGF, and IL-17 signaling pathways in cancer [72,73,74]. Overall, these findings indicate that isoflavones present in soy-fermented foods might play a role in suppressing lung cancer via the mediation of these signaling pathways based on our network pharmacology analysis results.

The molecular docking analysis performed in this network pharmacology approach has provided valuable insights into the potential interactions between the isoflavones of soy-fermented food products and the identified target proteins of lung cancer. Through the docking simulations, we aimed to identify key interactions, binding affinities, and potential binding modes of the ligands with their respective target proteins. The ADMET and toxicity analysis conducted within the network pharmacology framework has offered critical insights into the pharmacokinetic and safety profiles of the lead compounds [75]. The identification of ligands with favourable ADMET properties and manageable toxicities represents a significant step towards the selection of potential therapeutic candidates [76]. Furthermore, simulations of molecular dynamics can reveal the dynamic interactions between drugs and target molecules, providing a deeper understanding of their binding mechanisms, their stability, and their interactions [77]. Therefore, the screening and binding stability between the isoflavones in soy-fermented food products and the central targets were analyzed in the present study. Therefore, the present study examined the screening and binding stability between isoflavones in soy-fermented food products and the central targets in order to determine their efficacy. The results showed that only daidzein showed good ADMET properties and did not show any PAINS patterns with relatively good binding activity with the target proteins. Further, the binding stability of the daidzein–MMP9 complex was determined, as MMP9 is considered to be the most important target proteins in the lung cancer metastasis. In order to obtain the highest affinity between the ligand–protein complex of daidzein–MMP9, the molecular dynamics analysis revealed that these complexes display a stable conformation in solvation in water at a temperature of 300 K and at 1 atmosphere pressure. This is in line with what has been shown in the docking analysis. During the MD simulation, hydrogen bonds were found to be formed in both protein–ligand complexes, which indicates that the interaction has a high level of affinity.

Further experiments, including the anticancer and anti-metastasis analysis of daidzein on lung cancer cells, should be conducted to confirm the network pharmacological results. The biological effects of daidzein include antioxidation, anti-inflammation, chemoprevention, and anticancer properties [78]. There have been reports that this compound exhibits anticancer properties against different types of cancer, including breast and ovarian cancers [79,80]. In terms of its chemical composition, daidzein is similar to mammalian estrogens, and it can serve two different purposes by substituting for or inhibiting the estrogen and estrogen receptor (ER) complex [81]. Thus, Daidzein protects against many diseases, particularly those related to estrogen regulation, including diabetes, breast cancer, osteoporosis, and cardiovascular disease [81]. MTT assays are commonly used to evaluate a drug’s cytotoxic potential [81]. Using an MTT assay, the obtained results indicated that daidzein was capable of exhibiting anticancer effects on human lung cancer cells after 24 h of treatment. In a study conducted on SKVO3 cells, daidzein demonstrated potent anticancer activity with an IC_50_ of 20 µM. Nevertheless, it exhibited relatively low activity against normal ovarian Moody cells, with an IC_50_ of 100 µM [82]. It is believed that metastasis, which is the spread of cancer cells from the primary tumor to distant sites, is one of the most difficult aspects of cancer treatment and the leading cause of death due to cancer [83]. Understanding and targeting the mechanisms underlying metastasis is crucial to developing effective anti-metastatic therapies [84]. This study therefore further investigated the anti-metastasis potential of daidzein and discussed its implications for cancer therapy. The results of our study demonstrated a promising anti-metastatic effect of daidzein in lung cancer cells. Both in wound-healing assays and invasion assays, the treatment of lung cancer cells with daidzein significantly reduced cancer cell migration and invasion. 

A key mechanism underlying the anti-metastatic potential of daidzein could be its ability to inhibit the estrogen signaling pathway. It is believed that daidzein can provide a dual-directional purpose by providing a substitute for or hindering of estrogen as well as the estrogen receptor (ER) complex due to its similar chemical composition to mammalian estrogens [82]. Consequently, daidzein appears to be able to provide protective effects against a wide range of diseases, especially those that are connected to the control of estrogen, such as breast cancer, lung cancer, osteoporosis, diabetes, and cardiovascular disease [82]. Research has shown that estrogen signaling pathways may contribute to lung cancer development. Estrogen may be involved in lung cancer progression or initiation based on gender differences in lung cancer presentation [83]. A549 cells and lung cancer tissues have been shown to be upregulated by estrogen through the IGF-1R signaling pathway [84]. By activating the MEK/ERK signaling pathway, estrogen promotes lung cancer cell migration in addition to upregulating OPN expression [64]. Furthermore, it has been found that estrogen can promote the metastasis of non-small cell lung cancer (NSCLC) through the estrogen receptor β (ERβ)-mediated invasiveness-associated upregulation of matrix metalloprotease protein [85]. There are a number of mechanisms that contribute to estrogen’s promotion of lung cancer, and ER and IGF-1R are promising targets for combination therapy against lung cancer. MMP-9 is a protein that promotes metastasis and angiogenesis through extracellular matrix decomposition. It has been found to be involved in the estrogen signaling pathway. According to one study, estradiol induces MMP-9 expression in ERα-positive breast cancer cells via PELP1-mediated membrane-initiated signaling [86]. In a recent study, glutamic acid-, proline-, and leucine-rich protein 1 (PELP1) was found to be a novel ER coregulator, which has shown distinct characteristics from other ERα coregulators, and has recently been shown to play a role in the metastasis of several types of cancer [86]. According to the results of the study, estrogen-induced MMP-9 expression might be mediated through PI3K/Akt signaling pathways that are mediated by PELP1 in ER-positive breast cancer cells [86]. Overall, it was concluded that daidzein may have the potential to act as an active ingredient in soy-fermented food products in fighting cancer; however, it will be necessary to conduct further experiments to determine if it is the most significant active component in these products.

Thus, network pharmacology has proven to be an effective method of identifying the active ingredients in functional foods. It also identifies their molecular mechanisms of action. As a result of our study, there has been the first sustained evidence produced indicating that the use of fermented soy products, with the main component being daidzein, has proven to be beneficial in the treatment of cancer, specifically lung cancer. In light of our findings, further research into the mechanism of action of daidzein in treating lung cancer will certainly pave the way for the future.

## 5. Conclusions

As a whole, the present comprehensive study utilized an integrated approach that combines network pharmacology, molecular docking, molecular dynamics simulations, and rigorous in vitro validation in order to determine how soy-fermented food products affect lung cancer and the potential active components and intricate mechanistic pathways involved. Through this multifaceted analysis, we have successfully identified key bioactive compounds and unveiled the underlying interactions within cellular processes that contribute to the observed therapeutic effects. Our findings underscore the promising role of different soy-fermented food products as a valuable resource in the battle against lung cancer. The synergy of computational predictions and experimental validation has provided a robust foundation for understanding the intricate molecular mechanisms driving the anticancer potential of these products. This research not only contributes to the scientific understanding of the beneficial effects of soy-fermented food products but also offers a roadmap for future studies and potential therapeutic developments. As we continue to advance our knowledge in the field of integrative cancer research, the insights gained from this study pave the way for further exploration and optimization of isoflavones, potentially leading to the development of novel treatments or complementary strategies for lung cancer management. Our multidisciplinary approach exemplifies the power of combining computational and experimental methodologies to unravel complex biological phenomena, opening new avenues for innovative cancer therapeutics.

## Figures and Tables

**Table 1:** Information about the isoflavones of soy-fermented products along with their canonical smiles.

Sr. No.	Compound Name	MW	MF	Canonical Smile
1	Daidzein	254.24 g/mol	C15H10O4	C1=CC(=CC=C1C2=COC3=C(C2=O)C=CC(=C3)O)O
2	Genistein	270.24 g/mol	C15H10O5	C1=CC(=CC=C1C2=COC3=CC(=CC(=C3C2=O)O)O)O
3	Glycitein	284.26 g/mol	C16H12O5	COC1=C(C=C2C(=C1)C(=O)C(=CO2)C3=CC=C(C=C3) O)O
4	Malonylgenistin	518.4 g/mol	C24H22O13	C1=CC(=CC=C1C2=COC3=CC(=CC(=C3C2=O)O)OC4C (C(C(C(O4)COC(=O)CC(=O)O)O)O)O)O
5	Genistin	432.4 g/mol	C21H20O10	C1=CC(=CC=C1C2=COC3=CC(=CC(=C3C2=O)O)OC4C (C(C(C(O4)CO)O)O)O)O
6	Malonyldaidzin	502.4 g/mol	C24H22O12	C1=CC(=CC=C1C2=COC3=C(C2=O)C=CC(=C3)OC4C(C (C(C(O4)COC(=O)CC(=O)O)O)O)O)O

**Table 2:** List of genes with topological parameters (degree > 10).

Sr. No.	Genes	Degree	Subgraph	Betweenness	Closeness
1	TP53	93	1.70309E+16	1810.2233	0.30588236
2	AKT1	85	1.50395E+16	1209.3981	0.30023095
3	ALB	84	1.45701E+16	1478.0452	0.29885057
4	EGFR	79	1.42350E+16	767.9312	0.29411766
5	HSP90AA1	74	1.33435E+16	759.32733	0.29213482
6	TNF	74	1.30945E+16	661.356	0.29147983
7	HRAS	74	1.35786E+16	549.0256	0.29082775
8	SRC	72	1.37217E+16	405.83185	0.2888889
9	CASP3	71	1.36318E+16	412.17584	0.29017857
10	ESR1	70	1.25796E+16	633.2289	0.28761062
11	IGF1	61	1.10979E+16	277.7639	0.2832244
12	MMP9	59	1.03265E+16	269.15085	0.28199565
13	PTGS2	56	9.70134E+15	210.61162	0.2801724
14	RHOA	52	8.16216E+15	242.6943	0.27542374
15	BCL2L1	50	9.06884E+15	108.008354	0.2760085
16	ANXA5	50	9.48677E+15	84.69625	0.2760085
17	PPARG	49	7.89432E+15	152.48575	0.27542374
18	MDM2	47	7.67214E+15	143.88704	0.27310926
19	MMP2	47	7.70540E+15	102.22551	0.2736842
20	MAPK8	45	7.24961E+15	159.74908	0.2725367
21	CAT	44	5.77340E+15	371.0654	0.2736842
22	MAPK14	44	6.64498E+15	191.85359	0.2725367
23	KDR	43	6.42202E+15	77.25879	0.26915115
24	IGF1R	42	7.03299E+15	61.846313	0.27083334
25	SIRT1	42	6.57843E+15	140.43245	0.27139875
26	MAP2K1	39	6.27801E+15	48.690422	0.26804122
27	JAK2	39	5.92678E+15	45.057877	0.26639345
28	GSK3B	38	5.85577E+15	62.118233	0.26859504
29	IL2	37	5.63596E+15	53.313896	0.26859504
30	MET	37	5.14777E+15	47.117413	0.26530612
31	PTPN11	37	4.66587E+15	83.82157	0.26694044
32	MCL1	36	5.70959E+15	23.01386	0.26530612
33	KIT	36	4.97521E+15	37.521946	0.26530612
34	HMOX1	36	3.76490E+15	423.8162	0.26804122
35	AKT2	34	4.84180E+15	38.98536	0.26530612
36	XIAP	34	4.75574E+15	66.42771	0.26639345
37	RAF1	32	3.72559E+15	44.643333	0.26262626
38	CDK2	31	3.69572E+15	63.396782	0.2631579
39	TERT	31	3.84351E+15	67.03833	0.2631579
40	PARP1	31	4.07886E+15	32.731937	0.26476577
41	ABL1	31	3.41464E+15	43.111977	0.2615694
42	SERPINE1	30	3.35331E+15	46.154045	0.2636917
43	GSTP1	29	1.44186E+15	219.61592	0.26584867
44	SOD2	28	2.63405E+15	253.57841	0.2636917
45	MMP7	27	3.03206E+15	27.786367	0.26052105
46	ABCB1	27	2.60193E+15	63.017616	0.26262626
47	PRKCA	26	2.65481E+15	40.49914	0.25948104
48	ERBB4	25	2.46818E+15	16.559324	0.25742576
49	ACE	25	2.32904E+15	53.887497	0.2615694
50	ESR2	25	2.77889E+15	89.329414	0.26052105
51	CDK6	25	2.86819E+15	13.577173	0.26
52	PLAU	24	2.59795E+15	15.441549	0.25948104
53	NQO1	24	1.84276E+15	101.50741	0.26052105
54	MMP1	24	2.40993E+15	14.511479	0.26
55	TYMS	24	7.98944E+14	325.19824	0.25896415
56	FGFR1	23	2.36267E+15	10.2922325	0.25844932
57	IGFBP3	22	2.32580E+15	7.1022153	0.2579365
58	NOS2	22	2.14672E+15	16.07283	0.26052105
59	RAC1	22	1.68039E+15	21.24138	0.2524272
60	ABCG2	22	1.74603E+15	37.594463	0.26
61	AHR	22	1.66178E+15	42.12418	0.26
62	EIF4E	20	2.10336E+15	2.9916174	0.25641027
63	ALK	20	1.55143E+15	24.844717	0.2579365
64	PIK3CG	19	1.76235E+15	3.1658723	0.25390625
65	PLK1	19	1.42448E+15	19.416132	0.256917
66	GART	18	4.12615E+14	142.35718	0.25490198
67	LGALS3	18	1.53411E+15	33.23837	0.25742576
68	BRAF	18	1.21536E+15	9.635091	0.2534113
69	CTNNA1	17	9.45736E+14	9.677067	0.24667932
70	VDR	17	1.24125E+15	38.346687	0.25540274
71	ADAM17	17	1.03272E+15	27.434246	0.25291827
72	CYP1B1	17	5.92961E+14	53.069645	0.25291827
73	ARG1	17	8.11169E+14	160.89526	0.25390625
74	AURKA	17	1.21561E+15	13.74725	0.2534113
75	FGFR2	17	1.17520E+15	2.511399	0.2504817
76	RHEB	17	1.20739E+15	4.2154365	0.2524272
77	CSK	16	1.20519E+15	3.0542948	0.2504817
78	GSTM1	16	5.24847E+14	38.11805	0.2559055
79	TGFBR2	16	1.32542E+15	3.1019523	0.25440314
80	AXL	16	1.19384E+15	5.4183025	0.2514507
81	CYP19A1	16	1.35939E+15	3.7160077	0.25540274
82	SERPINA1	15	5.03674E+14	47.63756	0.24952015
83	DHFR	15	5.34485E+14	81.25459	0.25641027
84	CA9	15	1.04730E+15	46.289696	0.25390625
85	BCL2	15	1.15746E+15	1.0431849	0.25096524
86	AHCY	15	8.65762E+13	173.01955	0.24436091
87	CXCR2	14	1.02009E+15	4.7516828	0.2534113
88	TPI1	14	1.48572E+14	95.91854	0.2524272
89	MMP13	14	1.00270E+15	1.3570788	0.2524272
90	EPHA2	14	9.88097E+14	2.7505817	0.25096524
91	ELANE	13	5.21488E+14	5.72029	0.24809161
92	ALOX5	12	5.73864E+14	11.384069	0.251938
93	ABCC1	12	6.35524E+14	2.7090664	0.25390625
94	PRKCE	12	6.03043E+14	1.6471758	0.24574669
95	PON1	11	2.56962E+14	11.408818	0.24528302
96	TK1	11	7.71143E+13	59.961258	0.24436091
97	MMP12	10	3.26467E+14	3.6299863	0.24436091
98	None	10	1.69141E+14	23.737284	0.24856597
99	TTR	10	1.84150E+14	28.287405	0.24574669
100	MAPKAPK2	9	4.17365E+14	1.1214042	0.24904214
101	GSTM2	9	2.13918E+14	3.2141564	0.24856597
102	DAPK1	9	3.42158E+14	4.8307686	0.24809161
103	RARB	9	1.69496E+14	12.325137	0.24856597
104	KIF5B	9	2.27112E+14	5.4027066	0.23853211
105	None	8	1.54169E+14	28.305624	0.24714829
106	CRYZ	8	5.75053E+13	16.664522	0.22530329
107	PPIA	8	3.42044E+14	1.9932245	0.24856597
108	CES1	7	1.05845E+14	15.055967	0.24074075
109	IMPDH2	7	2.41610E+13	35.958282	0.24074075
110	FHIT	7	1.66404E+14	2.121033	0.24482109
111	TNK2	7	3.15551E+14	0.51937443	0.24761905
112	GPI	6	1.24437E+13	3.1605623	0.2249135
113	None	6	3.15992E+13	44.775955	0.24253732
114	CDA	6	2.41221E+13	32.566826	0.2416357
115	GC	6	2.50051E+13	2.7650793	0.23423423
116	SHMT1	6	6.31168E+12	1.602381	0.21630615
117	THRB	6	6.41589E+13	7.0238786	0.24118738
118	CFB	6	4.76834E+13	1.9542947	0.23593466
119	AZGP1	5	1.72523E+13	0	0.23339318
120	PLA2G2A	5	1.38081E+14	0.7182914	0.24299066
121	SLC6A3	4	1.00602E+14	0.13263159	0.24390244
122	AKR1C1	4	1.99457E+13	0.3605042	0.2184874
123	UCK2	4	1.33363E+12	1.3484849	0.20866774
124	DUSP3	4	7.62479E+13	0.08695652	0.23423423
125	SRM	3	1.14269E+12	3.9150116	0.21207178
126	NQO2	2	2.88130E+12	0	0.21276596
127	DOT1L	2	2.71917E+13	0	0.23593466
128	CA12	2	1.26821E+13	0	0.22569445
129	ATOX1	2	2.22952E+12	0.44444445	0.21416804
130	TAP1	0	1.00000E+00	0	0.007633588
131	ISG20	0	1.00000E+00	0	0.007633588

**Table 3:** List of compounds with topological parameters.

Sr. No.	Compound	Degree	Subgraph	Betweenness	Closeness
1	Genistin	104	4.68021088	3297.1821	0.6974359
2	Genistein	102	4.27065824	4020.0435	0.6834171
3	Malonylgenistin	99	4.34833952	2831.665	0.6634147
4	Malonyldaidzin	99	4.34633984	2870.7095	0.6634147
5	Glycitein	95	3.95949600	3162.9502	0.6384977
6	Daidzein	86	3.36311648	2407.4497	0.5887446

**Table 4:** ADME analysis of isoflavones in soy-fermented products.

Sr. No.	Compound	TPSA	C Log Po/w	GI Absorption	BBB Permeant	Lipinski Rule	PAINS #Alerts	Drug-Likeness
1	Daidzein	70.67	2.24	High	Yes	Yes	0	Yes
2	Genistein	90.9	2.04	High	No	Yes	0	Yes
3	Genistin	79.9	2.3	High	No	Yes	0	Yes
4	Glycitein	213.42	0.13	Low	No	No	0	No
5	Malonyldaidzin	170.05	0.35	Low	No	Yes	0	Yes
6	Malonylgenistin	193.19	0.22	Low	No	No	0	No

**Table 5:** Toxicity analysis of isoflavones in soy-fermented products.

Sr. No.	Compound	LD_50_	Hepatotoxicity	Carcinogenicity	Immunotoxicity	Mutagenicity	Cytotoxicity
1	Daidzein	2430 mg/kg	Inactive	Inactive	Inactive	Inactive	Inactive
2	Genistein	2500 mg/kg	Inactive	Inactive	Inactive	Inactive	Inactive
3	Genistin	2500 mg/kg	Inactive	Inactive	Inactive	Inactive	Inactive
4	Glycitein	2500 mg/kg	Inactive	Inactive	Inactive	Inactive	Inactive
5	Malonyldaidzin	5000 mg/kg	Inactive	Inactive	Inactive	Inactive	Inactive
6	Malonylgenistin	5000 mg/kg	Inactive	Inactive	Inactive	Inactive	Inactive

**Table 6:** The residues in the target proteins that interact with the isoflavones in soy-fermented products in their best-fitting pose.

Sr. No.	Protein	Receptor–Ligand	Interaction Type	Distance
1	ALB	A:ARG186:HN—N:UNK1:O	Conventional Hydrogen Bond	2.89731
		A:LEU115:CD1—N:UNK1	Pi–Sigma	3.99866
		A:TYR138—N:UNK1	Pi–Pi T-shaped	5.2795
		A:TYR161—N:UNK1	Pi–Pi T-shaped	5.16975
		N:UNK1—A:LEU115	Pi–Alkyl	4.36943
		N:UNK1—A:ARG117	Pi–Alkyl	5.35004
		N:UNK1—A:LYS137	Pi–Alkyl	5.49534
		N:UNK1—A:LEU182	Pi–Alkyl	4.9687
		N:UNK1—A:ARG186	Pi–Alkyl	5.10462
2	ANXA5	A:ASN232:ND2—UNL1:O	Conventional Hydrogen Bond	3.0243
		UNL1:H—A:THR254:OG1	Conventional Hydrogen Bond	2.20903
		A:LYS108:NZ—UNL1	Pi–Cation	3.9575
		A:LYS108:NZ—UNL1	Pi–Cation	4.62916
		A:LYS108:HN—UNL1	Pi-Donor–Hydrogen Bond	3.56838
		A:GLN235:NE2—UNL1	Pi-Donor–Hydrogen Bond	3.97156
		A:GLU107:C,O;LYS108:N—UNL1	Amide–Pi Stacked	4.45834
		UNL1—A:LYS108	Pi–Alkyl	4.73111
		UNL1—A:LYS108	Pi–Alkyl	4.14464
3	CASP3	UNL1—A:LYS105	Pi–Alkyl	5.30293
		UNL1—A:ARG147	Pi–Alkyl	5.1608
		UNL1—A:ARG147	Pi–Alkyl	4.14092
		UNL1—A:LYS105	Pi–Alkyl	4.38979
4	HRAS	A:GLY15:HN—UNL1:O	Conventional Hydrogen Bond	2.93639
		UNL1:H—A:ASP119:OD1	Conventional Hydrogen Bond	2.16104
		UNL1:H—A:TYR32:O	Conventional Hydrogen Bond	2.18025
		A:ASP30:CA—UNL1:O	Carbon–Hydrogen Bond	3.48989
		A:LYS117:NZ—UNL1	Pi–Cation	4.71779
		A:PHE28—UNL1	Pi–Pi T-shaped	4.82189
		UNL1—A:ALA18	Pi–Alkyl	4.3544
		UNL1—A:ALA18	Pi–Alkyl	4.95631
		UNL1—A:ALA18	Pi–Alkyl	5.43033
		UNL1—A:LYS117	Pi–Alkyl	4.23559
		UNL1—A:ALA146	Pi–Alkyl	5.42915
		UNL1—A:LYS147	Pi–Alkyl	5.43249
5	HSP90AA1	UNL1:H—A:ASP93:OD2	Conventional Hydrogen Bond	2.35485
		A:LEU107:CD2—UNL1	Pi–Sigma	3.88062
		A:LEU107:CD2—UNL1	Pi–Sigma	3.24717
		A:PHE138—UNL1	Pi–Pi Stacked	4.22964
		A:PHE138—UNL1	Pi–Pi Stacked	5.49423
		UNL1—A:VAL186	Pi–Alkyl	5.23903
6	MMP9	B:LEU418:CD1—UNL1	Pi–Sigma	3.69972
		B:THR426:CG2—UNL1	Pi–Sigma	3.62835
		B:HIS401—UNL1	Pi–Pi Stacked	5.87543
		B:HIS401—UNL1	Pi–Pi Stacked	4.30408
		B:TYR423—UNL1	Pi–Pi T-shaped	5.31933
		UNL1—B:LEU397	Pi–Alkyl	5.05191
		UNL1—B:VAL398	Pi–Alkyl	5.30563
		UNL1—B:ARG424	Pi–Alkyl	5.09726
7	PTGS2	B:ARG469:HN—UNL1:O	Conventional Hydrogen Bond	1.41545
		UNL1:H—B:GLY135:O	Conventional Hydrogen Bond	2.22526
		B:CYS47:HN—UNL1	Pi-Donor–Hydrogen Bond	2.67525
		B:LEU152:CD2—UNL1	Pi–Sigma	3.66252
		UNL1—B:CYS47	Pi–Alkyl	5.40895
		UNL1—B:PRO153	Pi–Alkyl	4.36592
		UNL1—B:VAL46	Pi–Alkyl	5.45328
		UNL1—B:CYS47	Pi–Alkyl	4.76239
		UNL1—B:PRO153	Pi–Alkyl	4.21059
8	SRC	UNL1:H—A:MET341:O	Conventional Hydrogen Bond	1.98724
		UNL1:C—A:THR338:OG1	Carbon–Hydrogen Bond	3.30631
		A:LEU393:CD1—UNL1	Pi–Sigma	3.64069
		A:TYR340—UNL1	Pi–Pi Stacked	5.95556
		UNL1—A:VAL281	Pi–Alkyl	5.42465
		UNL1—A:ALA293	Pi–Alkyl	3.82388
		UNL1—A:LEU273	Pi–Alkyl	5.40809
		UNL1—A:ALA293	Pi–Alkyl	4.88406
		UNL1—A:LEU393	Pi–Alkyl	4.89681
		UNL1—A:LYS295	Pi–Alkyl	4.75793
9	TNF	UNL1:H—A:LYS128:O	Conventional Hydrogen Bond	2.61045
		A:GLU127:OE1—UNL1	Pi–Anion	4.79338
		A:TYR87—UNL1	Pi–Pi Stacked	4.12668
		A:TYR87—UNL1	Pi–Pi Stacked	3.71965
10	TP53	A:ARG10:HH2—UNL1:O	Conventional Hydrogen Bond	2.76819
		UNL1:C—A:ASN17:O	Carbon Hydrogen Bond	3.39087
		UNL1—A:LYS20	Pi–Alkyl	5.48083

**Table 7:** The PASS webserver predicts the biological and structural properties of daidzein.

Sr. No.	Pa	Pi	Activity
1	0.967	0.002	Aldehyde oxidase inhibitor
2	0.960	0.001	Histidine kinase inhibitor
3	0.915	0.005	HIF1A expression inhibitor
4	0.887	0.014	Membrane integrity agonist
5	0.864	0.002	MMP9 expression inhibitor
6	0.850	0.005	Membrane permeability inhibitor
7	0.836	0.003	Antimutagenic
8	0.831	0.002	AR expression inhibitor
9	0.771	0.014	TP53 expression enhancer
10	0.756	0.001	RELA expression inhibitor
11	0.755	0.010	Apoptosis agonist
12	0.740	0.013	JAK2 expression inhibitor
13	0.712	0.007	HMOX1 expression enhancer

## Data Availability

All data generated or analyzed during this study are included in this article.
